# In Vitro Efficacy of Antibiotics Released from Calcium Sulfate Bone Void Filler Beads

**DOI:** 10.3390/ma11112265

**Published:** 2018-11-13

**Authors:** Phillip A. Laycock, John J. Cooper, Robert P. Howlin, Craig Delury, Sean Aiken, Paul Stoodley

**Affiliations:** 1 Biocomposites Ltd., Keele Science Park, Keele, Staffordshire ST5 5NL, UK; pl@biocomposites.com (P.A.L.); jjc@biocomposites.com (J.J.C.); cpd@biocomposites.com (C.D.); sa@biocomposites.com (S.A.); 2 National Institute for Health Research Southampton Respiratory Biomedical Research Unit, Southampton Centre for Biomedical Research, University of Southampton NHS Foundation Trust, Southampton SO17 1BJ, UK; rhowlin159@gmail.com; 3 National Centre for Advanced Tribology at Southampton (nCATS), Dept, Mechanical Engineering, University of Southampton, Southampton SO17 IBJ, UK; 4 Department of Microbial Infection and Immunity and Orthopedics, The Ohio State University, Columbus, OH 43210, USA

**Keywords:** calcium sulfate, antibiotics, release, zone of inhibition, biofilm

## Abstract

15 different antibiotics were individually mixed with commercially available calcium sulfate bone void filler beads. The antibiotics were: amikacin, ceftriaxone, cefuroxime, ciprofloxacin, clindamycin, colistamethate sodium, daptomycin, gentamicin, imipenem/cilastatin, meropenem, nafcillin, rifampicin, teicoplanin, tobramycin and vancomycin. The efficacy of specific released antibiotics was validated by zone of inhibition (ZOI) testing using a modified Kirby–Bauer disk diffusion method against common periprosthetic joint infection pathogens. With a subset of experiments (daptomycin, rifampin, vancomycin alone and rifampin and vancomycin in combination), we investigated how release varied over 15 days using a repeated ZOI assay. We also tested the ability of these beads to kill biofilms formed by *Staphylococcus epidermidis* 35984, a prolific biofilm former. The results suggested that certain antibiotics could be combined and released from calcium sulfate with retained antibacterial efficacy. The daptomycin and rifampin plus vancomycin beads showed antimicrobial efficacy for the full 15 days of testing and vancomycin in combination with rifampin prevented resistant mutants. In the biofilm killing assay, all of the antibiotic combinations showed a significant reduction in biofilm bacteria after 24 h. The exposure time was an important factor in the amount of killing, and varied among the antibiotics.

## 1. Introduction

Musculoskeletal infection represents a serious problem for patients, operating surgeons and the economic wellbeing of the healthcare system. Infection is a serious and debilitating complication of many clinical conditions and surgical procedures. Increasing resistance to current antibiotics is, according to the World Health Organization (WHO), “a problem so serious that it threatens the achievements of modern medicine” [1]. Reported in the Review of Antimicrobial Resistance, by 2050, “today’s already large 700,000 deaths every year would become an extremely disturbing 10 million every year, more people than currently die from cancer” [2]. The levels of antibiotics required to successfully manage infection are rising as bacterial resistance increases, to such a point where systemic levels required to be effective against the infection are increasingly toxic to the patient. An area gaining increasing interest is the combination and local release of antibiotics from suitable implantable materials. The benefits of local release at the site of infection are significant and include the ability to provide very high local levels of antibiotic, many times the minimal inhibitory concentration (MIC), with serum levels and associated toxicity remaining low. Reduced systemic administration may then be possible as, in addition to toxicity, exposure to antibiotics can lead to disruption of the normal human colonic flora and increased susceptibility to colonization and toxin production by *Clostridium difficile (C. difficile)*. *C. difficile* causes an inflammation of the colon and deadly diarrhea, and is one of the most common microbial causes of healthcare associated infections in USA hospitals [3]. One of the most adopted materials used in combination with antibiotics is poly-methyl methacrylate bone cement (PMMA) [3]. A number of commercially available antibiotic-loaded PMMA bone cements are shown in Table 1, where gentamicin is the most common combined antibiotic.

There is valid evidence to support the use of antibiotic-loaded PMMA in the form of spacers, bone cement for anchoring a prosthesis or as beads on a wire, however, there are still a number of significant disadvantages. PMMA is not absorbed in the body and needs a further operation for removal. In addition, the dead space following removal of the PMMA beads must be managed. Due to the way the antibiotic is contained within the material, following an initial burst release, it can continue to release sub-inhibitory levels over an extended time period increasing the risk of bacterial resistance [4,5,6]. PMMA is not suitable for thermosensitive antibiotics because of the high temperatures generated during curing. The combination of antibiotics with alternative biomaterials is now the subject of extensive investigation. A number of antibiotic-loaded biomaterials have received the European CE mark approval (Table 2) but have yet to be approved by the USA FDA (Food and Drug Administration). All these materials have advantages including good biocompatibility and drug/material compatibility but can have inherent disadvantages such as too rapid or inconsistent elution or, in the cases of calcium phosphates and composites, slow to incomplete absorption, which may present a potential nidus for infection. There is also a risk of damage to articulating surfaces from hard, non-absorbable biomaterials such as Hydroxyapatite (HA) with the migration of particles into the joint space, producing third-body wear and subsequent osteolysis [7].

One material with high potential is pure calcium sulfate. It is completely absorbed in the body and is biocompatible. Calcium sulfate has a long history of clinical use. Its first reported use in combination with a medicament was as early as 1892 when Dreesman and colleagues added 5% phenol (carbolic acid) solution to treat bone cavities as a result of tuberculosis osteomyelitis in long bones [8]. A typical residence time for calcium sulfate beads implanted in a contained bone defect is reported as 4 to 13 weeks [9,10,11,12]. In a soft tissue site and a site with high fluid exchange this time period may be considerably less (around three weeks) [13,14,15]. The antibiotic will elute predominantly over the first few days (burst release) followed by a gradual reduction in concentration as the calcium sulfate resorbs [16,17,18]. As there is a very small temperature rise on curing, mixing of heat labile antibiotics is possible [19,20]. With a well-established biocompatibility and resorption profile, calcium sulfate is increasingly being used in clinical practice for local antibiotic release [21,22,23,24].

Recent research has been carried out on the elution of specific antibiotics from calcium sulfate [16] but little work has been reported on the antimicrobial efficacy of the eluted antibiotic. In this study, investigations into the in vitro efficacy of the antibiotic(s) released from the calcium sulfate was carried out through zone of inhibition (ZOI) testing using the disk diffusion method against a range of susceptible pathogens, and compared to the published data according to the European Committee on Antimicrobial Susceptibility Testing (EUCAST) breakpoints [25] and Clinical and Laboratory Standards Institute (CLSI) M100 Standards [26]. In addition, in a subset of experiments, we used a repeated zone of inhibition assay and in vitro grown biofilms to expand on previous experiments with vancomycin and tobramycin [27], and to assess how the local elution of daptomycin and rifampicin alone and in combination with vancomycin may release over time and its efficacy of killing staphylococcal biofilms. Daptomycin has shown promise in treating patients with osteomyelitis or orthopaedic device infections when delivered systemically [28] and rifampicin has been shown to have good activity against staphylococcal biofilms but should be used in combination with other antibiotics due to concerns over resistance [29].

## 2. Materials and Methods

### 2.1. Preparation of Calcium Sulfate Beads

All bead/antibiotic combinations were prepared using a commercially available synthetic recrystallized calcium sulfate hemi-hydrate—CaSO_4_ ½H_2_O (SRCS) (Stimulan^®^ Rapid Cure, Biocomposites, Staffordshire, UK). This material is produced from pharmaceutical grade reagents without the addition of other excipients such as steric acid, to give a high purity, hydrophilic material having a physiological pH 10 cc packs of SRCS containing 20 g of calcium sulfate hemihydrate powder were used.

15 different antibiotics were selected based on published or ‘data on file’ clinical reports of their use with calcium sulfate [11,12,14,15,21,22,23,24] (Table 3). A number of bacteria were tested including *S. epidermidis* ATCC12228, *P. aeruginosa* NCTC 13437, *S. aureus* ATCC 6538, *Acinetobacter Baumanii* NCTC 134242, *S. aureus* 12493 MRSA, *E. faecalis* NCTC 12202, *P. acnes* NCTC 737 and *E. faecalis* NCTC 12201. For daptomycin, the maximum dose was referenced from the USP (United States Pharmacopeia) [30]. Sterile saline was used in the mixing of amikacin and daptomycin, replacing some or all of the mixing solution provided in the SRCS pack. The use of a 0.9% sodium chloride solution was found to speed up the setting reaction for these retarding antibiotics. The resultant paste, together with added antibiotic was pressed into hemispherical cavities, 6 mm diameter, in a flexible rubber mold where it was allowed to hydrate and set accordingly.

### 2.2. Zone of Inhibition Testing

Tryptone soya agar (TSA) plates were seeded with a 0.2 mL suspension of the relevant organism containing approximately 10^8^ CFU (Colony Forming Units)/mL. The plates were transferred to an incubator operating at 33 ± 2 °C for 30 min. The plates were then removed from the incubator and a single 6 mm antibiotic-loaded bead was placed on the surface of the agar. The plates were then incubated at 33 ± 2 °C for 24 h, after which time they were removed from the incubator and examined for any clear zones around the test sample. Zones were measured to the nearest mm where no obvious growth could be detected by the unaided eye. Samples were tested in triplicate and an average diameter was recorded. Zone diameters were compared to published data according to the European Committee on Antimicrobial Susceptibility Testing (EUCAST) breakpoints and Clinical and Laboratory Standards Institute (CLSI) M100 Standards [26] where applicable. It is important to note that this assay was not designed to determine breakpoint zones of the challenge strains against the various antibiotics. Breakpoint testing is performed using well-defined amounts of antibiotics loaded onto filter papers, which have well-characterized and reproducible release kinetics. Rather this assay was used to determine that the antibiotic was (1) released from the bead and (2) had retained antibiotic potency. Presence of a zone of inhibition was demonstrative that both these conditions were true. The known breakpoints from the standard method guidelines were provided as a reference guide.

### 2.3. Repeat Zone of Inhibition Testing

To assess how long the beads may release antibiotic, we used a modified Kirby–Bauer disk diffusion assay as previously reported [27]. *Staphylococcus aureus* NCTC 13143 EMRSA-16 (an MRSA strain) and *S. epidermidis* ATCC 35984, a prolific biofilm former was used as the challenge organisms. First, a lawn of bacteria was spread onto TSA plates using 50 µL of an overnight culture grown for 15 h at 37 °C. A single bead containing (in mg/5 cc pack SRCS) either (a) daptomycin (500 mg), (b) vancomycin (500 mg), (c) rifampicin (300 mg) or (d) a combination of vancomycin and rifampicin (500 mg + 300 mg) were placed on the agar plate using sterile forceps and incubated at 37 °C for 24 h. Zones of inhibition (ZOI) were measured and photographed, and then the beads were aseptically transferred onto a freshly prepared lawn of bacteria in a laminar flow hood as previously described [27]. This process was repeated each day until the ZOI was lost or the beads broke up. The area (cm^2^) of the ZOI was calculated using Image J (version 1.48) image analysis package. Area, rather than the diameter of the ZOI for these tests was reported to account for irregularities in the shape of the ZOI as the beads dissolved over time. We assessed the release and antimicrobial activity into agar rather than into an aqueous solution (which is another common method for measuring release kinetics) since we were interested in assessing the area of antimicrobial activity that a single bead might have in a diffusion-dominated environment such as what might be found adjacent to tissue.

### 2.4. Biofilm Killing Assay

*S. epidermidis* ATCC 35984 was used as the challenge organism. Overnight broth cultures were diluted in fresh Tryptone soya broth (TSB) to an optical density (OD) corresponding to 10^6^ cells/mL. 4 mL of the culture was added to each well of a 6 well plate (for viable cell counts) or tissue culture plates (MatTek, Corp.) (for confocal microscopy). Biofilms were grown for 72 h at 37 °C with a daily TSB nutrient exchange. Ten, 4.8 mm diameter beads were placed into each well plate and incubated at 37 °C for a further 24 h, 72 h or 1 week, with daily media changes. For cell counts, the wells were rinsed with 4 mL Hanks Buffered Salt Solution (HBSS) and a cell scraper was used to transfer the biofilm into 1 mL of HBSS. After scraping into 1 mL HBSS, the cells were vortexed using a lab bench vortexer for 20 s to homogenize the biofilm bacteria. A 10-fold serial dilution was plated onto tryptic soy agar (TSA; Sigma-Aldrich, St. Louis, MI, USA). Following incubation for 24 h at 37 °C, viable cell counts were performed, and the data expressed as CFU/cm^2^. Concurrently, confocal scanning laser microscopy (CSLM) of the biofilm was performed after 24h, 72 h and 1-week exposure to the beads. The biofilm was stained with Live/Dead Baclight (Invitrogen), which stains live cells green and dead cells red. After a 30 min incubation the plates were gently rinsed and observed using an inverted confocal laser scanning microscope (Leica DMI600 SP5, Wetzlar, Germany). The images were rendered using the freely downloadable NIH ImageJ. Each channel (green and red) was optimized for contrast using the “auto” setting. The channels were then merged using the “make composite” function. The individual z-sections in the stack were then compressed to show the full biomass in a 2-dimensional representation using “Z-project” with “sum slices”, and finally saved as a JPEG image.

### 2.5. Statistics

Viability CFU data were tested for normality using the Shapiro–Wilk test. Since the data were not normal (*p* > 0.05) they were compared using a Mann–Whitney rank sum test (Sigma-Plot, San Jose, CA, USA) for not normally distributed data, and a difference was considered significant when the *p* value was <0.05.

## 3. Results

### 3.1. Preparation of Calcium Sulfate Beads

Of the 15 antibiotics selected, 12 were in a lyophilized powder form. Where the powdered antibiotics were mixed with the SRCS by combining the powders together and then adding the aqueous mixing solution, all except ceftriaxone, daptomycin and imipenem/cilastatin allowed the SRCS to set hard but extended the setting time out from 4 min to a maximum of 20 min. For the powdered antibiotics that significantly delayed the setting time, the effect was reduced by hydrating the SRCS prior to the addition of the antibiotic thus initiating the calcium sulfate setting reaction whilst unloaded. This technique allowed the SRCS to set when ceftriaxone, daptomycin and imipenem/cilastatin were added. In addition, replacing the mixing solution with sterile saline reduced the set time even further with daptomycin. For the tobramycin and gentamicin liquid formulations, these were supplied in 2 mL vials, each containing 80 mg of antibiotic. For both these antibiotics, 3 vials were used to provide the required 6 mL for hydration of the SRCS, giving a dose of 240 mg. This was a limiting factor in the maximum dose, which may be combined. Two of the liquid antibiotic formulations; amikacin and clindamycin, were commercially provided in volumes of 2 mL and 4 mL. Additional fluid was required to make the volume up to 6 mL, required to fully hydrate the SRCS. For these antibiotics, a quantity of the aqueous mixing solution was accurately added via a syringe to make the volume up to the required 6 mL. Neither of these antibiotic combinations would set hard when the aqueous mixing solution was used, therefore, sterile saline was investigated as an alternative. This method allowed for the production of fully hardened amikacin-loaded beads. The clindamycin, which is in the form of a phosphate, may chemically react with the calcium sulfate, precipitating out as an insoluble calcium phosphate. In addition, this formulation of clindamycin contained benzyl alcohol and EDTA (ethylenediaminetetraacetic acid) as excipients. The ability of SRCS to set hard with Clindamycin in the form tested here was not achieved, therefore was unable to be combined in this way and no microbiology data was obtained.

### 3.2. Zone of Inhibition Testing

Zone diameters for each antibiotic/SRCS bead combination are shown in Table 4. Where EUCAST clinical breakpoint data [31] or CLSI M100 data [26] were available, they were included for reference. SRCS beads mixed with rifampicin achieved zones which fell below the CLSI M100 breakpoint data for both *S. epidermidis* and a methicillin-resistant strain of *S. aureus*. Interestingly, SRCS beads mixed with ciprofloxacin had varying success, depending on the species of bacteria under investigation. Ciprofloxacin-loaded SRCS beads were tested against a range of Gram-negative species including *P. aeruginosa* and *Acinetobacter Baumanii*. The zones of inhibition recorded for these species, fell below the EUCAST and CLSI breakpoints for the respective bacteria (12 and 15 mm respectively) (Table 4). Conversely, when SRCS were mixed with ciprofloxacin and tested against a range of Gram-positive species, the SRCS-loaded beads were able to generate zones measuring 36 and 25 mm (for *S. epidermidis* and *S. aureus* respectively). The observed zones were greater than the EUCAST and CLSI breakpoint for these bacterial species tested against this antibiotic. All other antibiotic combinations produced a clear zone against susceptible species and where breakpoint data was available, exceeded the breakpoint diameter, demonstrating an above MIC elution concentration and maintained efficacy (Table 4). For the vancomycin SRCS beads with *Enterococcus faecalis*, two vancomycin resistant control strains were used. NCTC 12201 (VanA-type glycopeptide resistance, Erythromycin resistant) and NCTC 12202 (VanA-type glycopeptide resistance). The results demonstrated that the high burst release concentration could exceed the vancomycin MIC of these two strains which are reported to exceed 256 µg/mL [32]. EUCAST and CLSI breakpoints have not been published for these strains but the zones observed were larger than the published breakpoints for susceptible strains of *E. faecalis* (Table 4).

### 3.3. Repeat Zone of Inhibition Testing 

**Figure 1:**
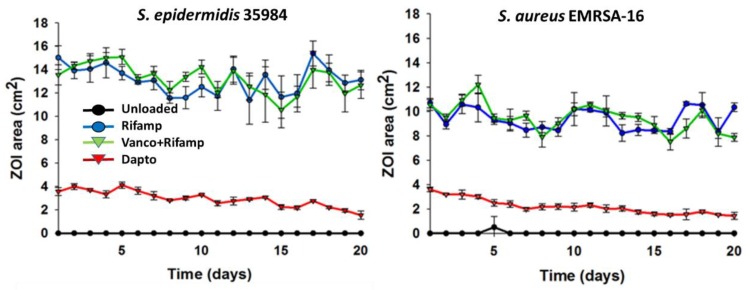
Repeated zone of inhibition (ZOI) of *S. aureus* NCTC 13143 EMRSA-16 and *S. epidermidis* ATCC 35984 Stimulan^®^ beads loaded with rifampicin, rifampicin and vancomycin or daptomycin. Assays were performed in triplicate and data expressed as the mean and 1SD.

In the case of beads tested against *S. aureus* NCTC 13143 EMRSA-16 strains, large ZOIs were maintained for 20 days in the case of rifampicin and rifampicin and vancomycin in the combination beads (Figure 1). In the case of daptomycin-loaded beads, the size of the ZOI was smaller (~4 cm^2^) but were also maintained for the 20 days duration. A similar trend was seen for beads tested against a *S. epidermidis* ATCC 35984 strain. 

**Figure 2:**
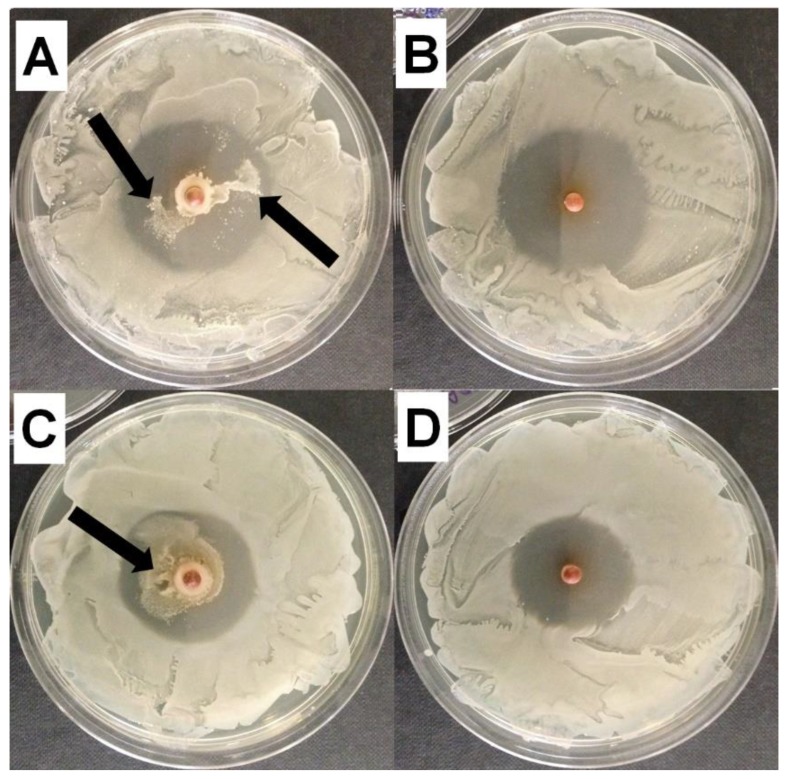
Representative image of the Zones of Inhibition (ZOI) observed with (**A**,**B**) *S. epidermidis* ATCC 35984 and(**C**,**D**) *S. aureus* NCTC 13143 EMRSA-16 at day 20 of rifampicin and vancomycin in combination, showing no evidence of resistant colonies (**B**,**D**) and rifampicin alone (**A**,**C**) showing potential resistant mutant colonies growing within the ZOI (black arrows).

At the end of the 20 day assay, suspected resistance was noted developing in the rifampicin-only loaded beads, as shown by an internal ring of viable bacteria within the ZOI (Figure 2A,C). This was not observed on plates that had been exposed to beads containing rifampicin and vancomycin in combination (Figure 2B,D).

### 3.4. Biofilm Killing Assay

**Figure 3:**
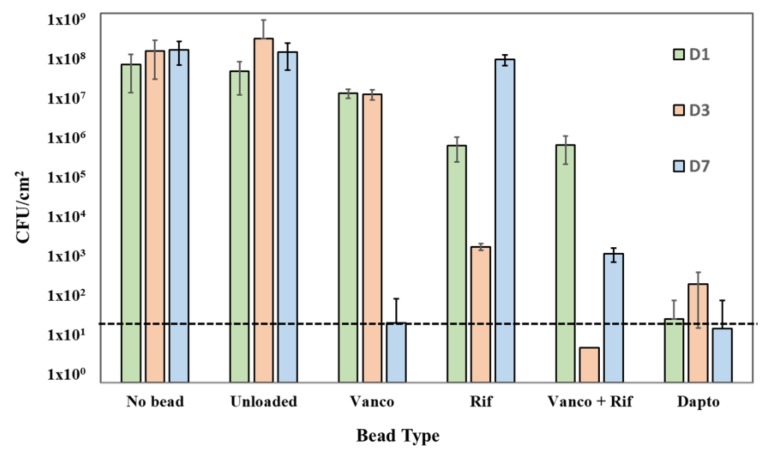
Effect of unloaded beads as well as vancomycin (Vanco), rifampicin (Rif), rifampicin and vancomycin in combination (Vanco + Rif) and daptomycin (Dapto) loaded beads on established *S. epidermidis* ATCC 35984 biofilms at contact times at days (D) 1,3 and 7. Dashed line is the detection limit. No beads were added as a positive control for biofilm growth. Mean and 95% CI (n = 3), *indicates statistically significant differences from the unloaded beads (*p* < 0.05).

**Figure 4:**
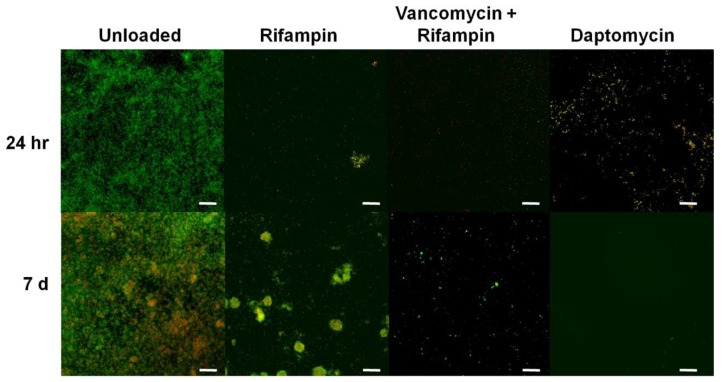
Representative CSLM images showing *S. epidermidis* ATCC 35984 biofilm (live cells stained green and dead and membrane compromised cells stained red or yellow) following treatment for 24 h and 1 week with unloaded beads (negative control) and beads loaded with rifampicin (Rifampin), rifampicin and vancomycin, and daptomycin. Scale bars: 25 µm.

Beads loaded with rifampicin, and vancomycin and rifampicin in combination were able to achieve a 2-log reduction in CFU/cm^2^ at 24 h (Figure 3) with CSLM imaging showing a concurrent marked reduction in biofilm mass (Figure 4). After 72 h contact time, rifampicin alone achieved a 5-log reduction in CFU/cm^2^ (Figure 3) with the combination of vancomycin and rifampicin showing no growth on the plate. CSLM imaging showed almost complete removal of the biofilm with only a few single cells remaining (Figure 4). However, at one week, regrowth was noted in rifampicin and rifampicin and vancomycin treatment groups and reformation of microcolonies noted with CSLM imaging (Figure 4). Additionally, the combination of rifampicin and vancomycin demonstrated a 3-log regrowth relative to CFU/cm^2^ data at 72 h suggesting that the regrowth may have been due to some cells that were rendered viable but nonculturable at 72 h. Daptomycin-loaded beads resulted in an approximate 7-log reduction in CFU/cm^2^ relative to unloaded beads after 24 h contact time with mature *S. epidermidis* biofilms (Figure 3). There was little further change over the next seven days. CSLM imaging corroborated the CFU data showing a significant reduction in biofilm and total surface coverage observed at day 7 relative to unloaded beads (Figure 4). Interestingly, in the biofilm exposed to the rifampicin only beads, after 7 days there was the appearance of larger aggregates, which had both live (green) and dead (red) cells suggesting the proliferation of resistant mutants. In the control most of the cells were still live (green) however, there were patches of red (dead or compromised) cells suggesting that the cells may have been undergoing starvation due to nutrient limitation. In the biofilms exposed to the combination of vancomycin and rifampicin there was a sparse covering of cells, which were mainly live, while in the daptomycin-exposed biofilm there were very few cells and these were only very faintly stained green (Figure 4).

## 4. Discussion

With the exception of clindamycin, the calcium sulfate in combination with the antibiotics tested, set hard, maintaining its function as a void filler. All antibiotics used in this study were IV formulations. Antibiotic capsules and tablets contain different excipients to IV formulations and these may create additional risks if administered locally. Ceftriaxone, although included here, is contraindicated when mixing with a calcium-containing product such as Ringer’s solution, Hartmann’s solution or parenteral nutrition containing calcium. The reason for this contraindication is the risk of crystalline precipitates forming in the lungs and kidneys. Although this risk is for IV administration, the risks should be considered if used in combination with calcium sulfate in this application and warrants further investigation. The ability of SRCS or any biomaterial to mix and set with antibiotics, and demonstrate release and potency alone or in combination in in vitro laboratory studies, does not imply safety or efficacy in clinical use, and in vitro results may not be indicative of clinical performance.

A number of independent studies have shown that calcium sulfate beads may be used in revision arthroplasty for PJI (Periprosthetic Joint Infection), with favorable outcomes [21,33,34]. However, their use is not without risk. One potential risk is transitory hypercalcemia in vivo and this has been reported in literature, [15,21] although only when the material has been implanted in soft tissue sites and in larger volumes. More recent studies however, have shown that there is no significant difference in incidences of hypercalcemia when compared with other antibiotic-loaded materials [35].

This study demonstrates that a wide range of antibiotics can be incorporated into the SRCS and retain potency against a wide range of common bacterial pathogens associated with PJI using an *in vitro* zone of inhibition assay. Where EUCAST or CLSI breakpoints were available for specific antibiotics against susceptible pathogens, the same antibiotics when eluted from the SRCS all maintained their antimicrobial activity with the exception of ciprofloxacin and rifampicin. This assay is a relatively simple test which can be used to assess whether antibiotics as monotherapy or in combination, which (a) are currently used to treat orthopedic infections systemically or (b) show promise in in vitro studies also have potential to be eluted locally by SRCS made up as beads or in other forms. Although the standardized Kirby–Bauer disc diffusion method is used as a quick and convenient method of assessing the susceptibility of a given organism to a given antibiotic [36], modifications of the assay allow it to be used to assess the release and maintenance of potency from materials other than filter paper, such as how we have done with materials such as SRCS, and PMMA as detailed in previous studies [27]. The results for ciprofloxacin against *Pseudomonas aerugonisa* (NCTC 13437) and *Acinetobacter baumannii* (NCTC 134242) showed a smaller zone than the breakpoint data for Pseudomonas spp. (Table 4). This may be due to the susceptibility of the specific species or other factors such as compatibility with the SRCS or reduced elution.

The quantity of each antibiotic held in the disks used in the EUCAST or CLSI M100 may differ to the quantity of antibiotic in the SRCS beads, therefore, correlation to the clinical breakpoints should not be inferred and these values are included for reference and demonstration of susceptibility only. Future work should focus on testing the release of antibiotics from SRCS as per the guidance of EUCAST or CLSI in order to make a more direct comparison of the efficacy of released antibiotic from SRCS beads. 

We further modified the method to assess the longevity of release in vitro by placing SRCS on new spread plates daily. The ZOI for daptomycin started at approximately 4 cm^2^ and slowly reduced to 2 cm^2^ after 20 days of the experiment for both the EMRSA and *S. epidermidis* challenge strains. This behavior was very similar to that seen with vancomycin and tobramycin assessed previously [27]. Interestingly the SRCS beads containing vancomycin and rifampicin or rifampicin alone had much greater ZOIs of between 14 cm^2^ and 10 cm^2^ against EMRSA and *S. epidermidis,* respectively. These remained relatively constant over the full 20 days of testing. 

Rifampicin has been noted to have “excellent activity on adherent staphylococci” [37] and biofilms [29]. However, since it is known that there is rapid emergence of resistance when used in monotherapy [38] it is used in combination with other antibiotics. Indeed, in our assay we noted growth within the ZOI of the beads loaded with rifampicin alone (Figure 2). Since the plates were spread freshly every day it was possible that the growth came from bacteria carried over with the bead that had been exposed to rifampicin for an extended period. Interestingly, the beads with both vancomycin and rifampicin showed no evidence of the emergence of resistance. However, to more fully assess the potential for the development of resistance with vancomycin and rifampicin, more stringent studies are required. The *S. epidermidis* biofilm killing assay showed different patterns of killing with the various antibiotics. Vancomycin-alone only caused an approximate 1-log reduction after 1 and 3 days exposure but by 7 days there was a 6-log reduction illustrating the importance of prolonged exposure to high concentrations of antibiotics when treating biofilms. Rifampicin alone was more effective, showing an approximate 2-log reduction after 1 day and 5-log reduction after 3 days, however, there was significant regrowth at day 7. The confocal image showed large cell clusters had formed suggesting the emergence of resistant mutants within this time period (Figure 4). Rifampicin and vancomycin in combination reduced the biofilm to below detectable levels after 3 days (>7-log reduction) but there was a “bounce” back at day 7 to an approximate 5-log reduction compared to the control unloaded beads. Although there was no evidence of new colony formation from the confocal data, more work needs to be done to assess the potential for the emergence of resistance with this combination. However, a combination of vancomycin and rifampicin has been shown to be more effective than vancomycin alone in treating PJI in a mouse model [39], and the local release of rifampicin from SRCS in combination with other antibiotics to treat orthopedic biofilm infections shows promise. Although daptomycin resulted in the lowest ZOI in the diffusion assay, it caused the greatest reduction in biofilm (approximately 6 logs) after only 1-day exposure, however, little further reduction was seen after the longer exposure periods.

## 5. Conclusions

The use of locally released antibiotics from synthetic recrystallized calcium sulfate may offer significant benefits in the management of surgical site infections. The ability to combine different antibiotics could enable a therapy tailored to the offending pathogens. The exposure time, type of antibiotic(s) released from SRCS and the challenge bacterial strain can all influence the kinetics of biofilm killing and thus, in vitro results with one set of conditions should not be over generalized.

## Figures and Tables

**Table 1:** Antibiotic-loaded PMMA cements.

Name	Antibiotic	Manufacturer	CE Mark	FDA 510 (k)
CEMEX^®^ Genta	CEMEX^®^ Genta	CEMEX^®^ Genta	Yes	K043403
Copal^®^ G + C	Gentamicin, Clindamycin	Heraeus GmbH, Hanau, Germany	Yes	No
Copal^®^ G + V	Gentamicin, Vancomycin	Heraeus GmbH, Hanau, Germany	Yes	No
Palacos^®^ R + G	Gentamicin	Heraeus GmbH, Hanau, Germany	Yes	K031673
Palamed^®^ G	Gentamicin	Heraeus GmbH, Hanau, Germany	Yes	K050855
Smartset GHV	Gentamicin	Depuy Orthopaedics, Rosemont, IL, USA	Yes	K033563
Simplex^®^ P	Tobramycin	Stryker, Kalamazoo, MI, USA	Yes	K014199
VancogeneX^®^	Vancomycin, Gentamicin	Tecres S.P.A, Verona, Italy	Yes	No

**Table 2:** Antibiotic-loaded biomaterials (CE marked).

Name	Composition	Antibiotic	Manufacturer
Cerament™ G	Calcium sulfate/HA	Gentamicin	Bonesupport AB, Lund, Sweden
Cerament™ V	Calcium sulfate/HA	Vancomycin	Bonesupport AB, Lund, Sweden
Collatamp^®^ G	Collagen	Gentamicin	EUSAPharma Ltd., Hemel Hempstead, UK
Herafill^®^ beads G	Calcium sulfate/Calcium carbonate	Gentamicin	Heraeus GmbH, Hanau, Germany
Osteoset^®^ T	Calcium sulfate	Tobramycin	Wright Medical Technology, Inc., Memphis, TN, USA

**Table 3:** Antibiotics tested in this study.

Antibiotic	Manufacturer
Amikacin Sulfate	Hospira Ltd., Maidenhead, UK
Ceftriaxone Sodium	Apotex Corporation, Toronto, ON, Canada
Cefuroxime	Stravencon, London, UK
Ciprofloxacin Hydrochloride	Medisca Inc., Las Vegas, NV, USA
Colistamethane Sodium	Sigma-Aldrich, Dorset, UK
Clindamycin (Dalacin C^®^ Phosphate)	Pfizer, Tadworth, UK
Daptomycin (Cubicin^®^)	Novartis, Basel, Switzerland
Gentamicin Sulfate	Hospira Ltd., Maidenhead, UK
Imipenem/Cilastatin (Zienam^®^)	Merck & Co., Inc, Kenilworth, NJ, USA
Meropenem Trihydrate	Fresenius Kabi Ltd., Cestrian, UK
Nafcillin Sodium	Sandoz, Princeton, NJ, USA
Rifampicin	Sigma-Aldrich, Dorset, UK
Teicoplanin (Targocid^®^)	Sanofi-Aventis, Guildford, UK
Tobramycin Sulfate	Hospira Ltd., Maidenhead, UK
Vancomycin Hydrochloride	Hospira Ltd., Maidenhead, UK

**Table 4:** Zones of Inhibition (ZOI) data with EUCAST clinical breakpoint tables v 6.0 and CLSI M100 breakpoints.

Antibiotic Conc. per 10cc Pack SRCS	Species	Zone Diameter (mm)	EUCAST/ CLSI M100	Zone Diameter Breakpoint (mm)		Note
				Susceptible ≥	Resistant ≤	
Amikacin 500 mg/2 mL	*S. epidermidis* (ATCC 12228)	30	EUCAST	22 ^a^	19 ^a^	^a^ Coagulase negative staphylococci
			CLSI	-	-	n/a
	*P. aeruginosa* (NCTC 13437)	20	EUCAST	18 ^c^	15 ^c^	^c^*Pseudomonas* spp.
			CLSI	15	12	^-^
Ceftriaxone 1 g	*S. aureus* (ATCC 6538)	47	EUCAST	-	-	n/a
			CLSI	-	-	n/a
	*S. epidermidis* (ATCC 12228)	31	EUCAST	-	-	n/a
			CLSI	-	-	n/a
Cefuroxime 1.5 g	*S. aureus* (ATCC 6538)	47	EUCAST	-	-	n/a
			CLSI	-	-	n/a
	*S. epidermidis* (ATCC 12228)	22	EUCAST CLSI	- -	- -	n/a n/a
	*P. aeruginosa* (NCTC 13437)	28	EUCAST	-	-	n/a
			CLSI	-	-	n/a
	*Acinetobacter Baumannii* (NCTC 134242)	22	EUCAST CLSI	- -	- -	n/a n/a
Colistamethane Sodium 400 mg	*S. aureus* (ATCC 6538)	9	EUCAST CLSI	- -	- -	n/a n/a
	*S. epidermidis* (ATCC 12228)	11	EUCAST CLSI	- -	- -	n/a n/a
	*P. aeruginosa* (NCTC 13437)	13	EUCAST CLSI	- -	- -	n/a n/a
Ciprofloxacin 1 g	*P. aeruginosa* (NCTC 13437)	17	EUCAST CLSI	25 ^c^ 21	22 ^c^ 15	^c^*Pseudomonas* spp. -
	*Acinetobacter Baumannii* (NCTC 134242)	15	EUCAST CLSI	21 ^d^ 21 ^d^	21 ^d^ 15 ^d^	^d^*Acinetobacter* spp. ^d^ *Acinetobacter* spp.
Ciprofloxacin 1 g	*S. epidermidis* (ATCC 12228) *S.aureus* (NCTC 12493) MRSA (NCTC 134242)	36 25	EUCAST CLSI EUCAST CLSI	20 ^b^ 21 ^b^ 20 ^b^ 21 ^b^	20 ^b^ 15 ^b^ 20 ^b^ 15 ^b^	^b^*Staphylococcus* spp. ^b^ *Staphylococcus* spp. ^b^ *Staphylococcus* spp. ^b^ *Staphylococcus* spp.
Daptomycin 1 g	*S. epidermidis* (ATCC 12228) E. faecalis * (NCTC 12202) *P. acnes* (NCTC 737) *P. aeruginosa* (NCTC 13437)	25 13.5 31 11	EUCAST CLSI EUCAST CLSI	- - - -	- - - -	n/a n/a n/a n/a
			EUCAST CLSI EUCAST CLSI	- - - -	- - - -	n/a n/a n/a n/a
Gentamicin 240 mg	*S. aureus* (ATCC 6538) *S. epidermidis* (ATCC 12228)	20 30	EUCAST CLSI EUCAST CLSI	18 15 ^b^ 22 ^a^ 15 ^b^	18 12 ^b^ 22 ^a^ 12 ^b^	- ^b^ *Staphylococcus* spp. ^a^ Coagulase negative staphylococci ^b^ *Staphylococcus* spp.
Imipenem & Cilastatin 500 mg	*S. aureus* (ATCC 6538) *S. aureus* (NCTC 12493) MRSA	58 49	EUCAST CLSI	- -	- -	n/a n/a
			EUCAST CLSI	- -	- -	n/a n/a
	*S. epidermidis* (ATCC 12228)	60	EUCAST CLSI	- -	- -	n/a n/a
Meropenem 1 g	*S. epidermidis* (ATCC 12228) *S. aureus* (NCTC 12493) MRSA	56 37	EUCAST CLSI	- -	- -	n/a n/a
			EUCAST CLSI	- -	- -	n/a n/a
	*P. aeruginosa* (NCTC 13437) *Acinetobacter Baumannii* (NCTC 134242)	28 22	EUCAST CLSI	24 ^c^ 19	18 ^c^ 15	^c^*Pseudomonas* spp. -
			EUCAST CLSI	21 ^d^ 18 ^d^	15 ^d^ 14 ^d^	^d^*Acinetobacter* spp. ^d^ *Acinetobacter* spp.
Nafcillin 1 g	*S. aureus* (ATCC 6538)	51	EUCAST CLSI	- 22 ^b^	- 21 ^b^	n/a ^b^ *Staphylococcus* spp. Cefoxitin (Oxicillin surrogate)
	*S. epidermidis* (ATCC 12228) *P. aeruginosa* (NCTC 13437)	57 13	EUCAST CLSI	- 22 ^b^	- 21 ^b^	n/a ^b^ *Staphylococcus* spp. Cefoxitin (Oxicillin surrogate)
			EUCAST CLSI	- -	- -	n/a n/a
Rifampicin 600 mg	*S. epidermidis* (ATCC 12228) *S. aureus* (NCTC 12493) MRSA	15 11	EUCAST CLSI	- 20 ^b^	- 16 ^b^	n/a ^b^ *Staphylococcus* spp.
			EUCAST CLSI	- 20 ^b^	- 16 ^b^	n/a ^b^ *Staphylococcus* spp.
Teicoplanin 400 mg	*S. aureus* (ATCC 6538) *S. aureus* (NCTC 12493) MRSA *S. epidermidis* (ATCC 12228)	18 26 23	EUCAST CLSI EUCAST	- - -	- - -	n/a n/a n/a
			CLSI EUCAST CLSI	- - -	- - -	n/a n/a n/a
Tobramycin 1.2 g	*S. aureus* (ATCC 6538) *S. epidermidis* (ATCC 12228)	19 28	EUCAST CLSI	18 -	18 -	- n/a
			EUCAST CLSI	22 ^a^ -	22 ^a^ -	^a^ Coagulase negative staphylococci n/a
Vancomycin 1 g	*S. aureus* (ATCC 6538) *S. epidermidis* (ATCC 12228) *E. faecalis* * (NCTC 12201) *E.faecalis* * (NCTC 12202)	21 23 17–18 12–13	EUCAST CLSI EUCAST CLSI	- - - -	- - - -	n/a n/a n/a n/a
			EUCAST CLSI EUCAST CLSI	12 ^e^ 17 ^e^ 12 ^e^ 17 ^e^	12 ^e^ 14 ^e^ 12 ^e^ 14 ^e^	^e^*Enterococcus* spp. ^e^ *Enterococcus* spp. ^e^ *Enterococcus* spp. ^e^ *Enterococcus* spp.

* Denotes Vancomycin Resistant Control Strains. (^a–e^) Represent the breakpoints for that particular species.
